# Remote olfactory assessment using the NIH Toolbox Odor Identification test and the brain health registry

**DOI:** 10.1371/journal.pone.0301264

**Published:** 2024-04-18

**Authors:** Cristina Jaén, Christopher Maute, Scott Mackin, Monica R. Camacho, Diana Truran, Rachel Nosheny, Michael W. Weiner, Pamela Dalton

**Affiliations:** 1 Monell Chemical Senses Center, Philadelphia, Pennsylvania, United States of America; 2 University of California San Francisco, San Francisco, California, United States of America; 3 Department of Veteran Affairs Medical Center, San Francisco, California, United States of America; 4 Department of Veterans Affairs Medical Center, Northern California Institute for Research and Education (NCIRE), San Francisco, California, United States of America; University of Cadiz: Universidad de Cadiz, SPAIN

## Abstract

**Background:**

Early identification of deficits in our ability to perceive odors is important as many normal (i.e., aging) and pathological (i.e., sinusitis, viral, neurodegeneration) processes can result in diminished olfactory function. To realistically enable population-level measurements of olfaction, validated olfaction tests must be capable of being administered outside the research laboratory and clinical setting.

**Aim:**

The purpose of this study was to determine the feasibility of remotely testing olfactory performance using a test that was developed with funding from the National Institutes of Health as part of a ready-to-use, non-proprietary set of measurements useful for epidemiologic studies (NIH Toolbox Odor ID Test).

**Materials and methods:**

Eligible participants older than 39 years and active (within 6 months) in the Brain Health Registry (BHR), an online cognitive assessment platform which connects participants with researchers, were recruited for this study. Interested participants were mailed the NIH Toolbox Odor ID Test along with instructions on accessing a website to record their responses. Data obtained from subjects who performed the test at home was compared to the normative data collected when the NIH Toolbox Odor ID Test was administered by a tester in a research setting and validated against the Smell Identification Test. The age-range and composition of the population ensured we had the ability to observe both age-related decline and gender-related deficits in olfactory ability, as shown in the experimental setting.

**Results:**

We observed that age-associated olfactory decline and gender-associated performance was comparable to performance on the administered test. Self-administration of this test showed the age-related loss in olfactory acuity, F(4, 1156)=14.564, p<.0001 as well as higher accuracy for women compared to men after controlling for participants’ age, F(1, 1160) = 22.953, *p* <.0001. The effect size calculated as Hedge’s g, was 0.41.

**Conclusion:**

These results indicate that the NIH Toolbox Odor ID Test is an appropriate instrument for self-administered assessment of olfactory performance. The ability to self-administer an inexpensive olfactory test increases its utility for inclusion in longitudinal epidemiological studies and when in-person testing is not feasible.

## Introduction

Our sense of smell provides information about our environment and is vital for critical functions such as food choice awareness of danger, and social functioning. Olfactory dysfunction affects quality of life through deleterious impacts on physical and mental health, as well as social relationships. Prior to the impact of COVID-19, decreased or absent olfactory function (hyposmia or anosmia, respectively) was estimated to afflict 3–20% of the population [1]. Despite this prevalence, many individuals with olfactory dysfunction are not aware of their problem until tested, particularly when smell loss occurs gradually, such as occurs with aging, beginning around the 5th decade of life [2]. There are multiple causes of olfactory disorders including: allergies, bacterial and viral infections, head injuries, sino-nasal disease, and environmental exposures to toxins and pollutants [3, 4]. Importantly, smell dysfunction is one of the hallmark “preclinical” signs of neurodegenerative diseases such as Alzheimers and Parkinsons [5–7]. Results from the longitudinal NHANES assessment of smell function revealed that olfactory dysfunction was one of the strongest predictors of 5-year mortality in older adults [8] as well as a predictor of dementia [9]. In fact, olfactory function appears to be a good indicator of general health [10, 11], thus emphasizing its importance as a clinical health assessment tool.

Measurement of olfactory ability in the clinical or research setting usually consists of odor identification, odor discrimination, and odor detection threshold tasks [12]. Odor identification tests consist of the presentation of a supra-threshold concentration of an odor to a subject nose orthonasally who must choose the appropriate odor name from several response options. The most common odor identification tests to date are the University of Pennsylvania Smell identification Test (UPSIT) [13], which is a scratch and sniff test with 40 microencapsulated odorants, and the Sniffin’ Sticks test, which is a set of 16 odors that are presented in liquid form using felt-pens [14]. While these are considered ‘gold-standard’ tests with normative data on many thousands of individuals, they are proprietary and thus the cost of usage in large studies can become prohibitive.

In addition, several of these tests require a trained administrator limiting its use for remote testing. Remote testing has seen more attention recently due to research reliance on it for studies during the COVID-19 pandemic. Olfaction loss, due to its symptomatic association with COVID-19, has especially been targeted for remote testing to damper the spread of disease by detection and understand the prevalence of long-term loss. The UPSIT, a remote-capable test, had previously been validated for remote testing and was utilized during the COVID-19 pandemic to signify alterations in the patient population [15]. Similar results have been shown in new remote chemosensory tests reaffirming remote and centralized testing may give similar results. No differences were seen in taste preferences or ratings of sugar solutions [16] nor overall sensory evaluations of products [17].

As a part of the larger NIH Toolbox initiative, the NIH Toolbox Odor ID Test was created to be “off the shelf”, brief, inexpensive, and suitable for use across the lifespan [18]. Similar to the UPSIT, the NIH Toolbox Odor ID Test is a “scratch-and-sniff” test that uses pictorial answers to identify odors and was originally designed with the intention of being administered with the participant and the tester in the same room. The adult version of the test consists of 9 odors that are microencapsulated and placed onto small individual cards. The goal of this study was to evaluate performance on this test when self-administered in the participant’s home. The main focus of the study was to determine the feasibility of remote unsupervised testing. We explored whether the mailing of the odor cards together would result in cross contamination of odors and whether unsupervised results obtained at home would replicate previous results obtained during tester administration.

## Methods

The study reported here was conducted in accordance with the Code of Ethics of the World Medical Association (Declaration of Helsinki) for experiments involving humans. It was approved by the University of Pennsylvania Institutional Review Board. All participants provided on line consent before providing demographic data and taking the olfactory test.

### Participants

Participants were recruited through the Brain Health Registry (BHR). The BHR is an online voluntary registry overseen by the University of California at San Francisco (UCSF), in which interested individuals report health information and complete standardized cognitive testing at regular intervals [19]. The aim of the BHR is to advance research aimed at Alzheimer’s and other brain disorders. Participants must be at least 18 years old to be a part of the registry. At the time of the study, the registry had 42,415 participants. About 74 % of the participants self-identified as female and 26% as male. The vast majority of the registered BHR participants (85%) were older than 39 years old. Participants aged 40 years or older were targeted for this study due to the potential for variability in olfactory performance in this cohort.

An a priori power analysis was conducted using G*Power version 3.1.9.7 [20] for sample size estimation, based on data from the normative study assessing performance across genders and the lifespan [18 in which the effect size was considered ‘moderate’]. With a significance criterion of α = .05 and power = .80, the minimum sample size needed with this effect size was *N* = 405. Thus, the obtained sample size of *N* = 1163 is more than adequate to test the study hypothesis." We included more interested and willing participants than needed in this preliminary study as we wished to follow up their performance and cognitive function at 5+ years intervals and needed to have sufficient participants still engaged and willing to participate.

### Procedure

Eligible, interested participants were emailed study information by the BHR study team. Eligible participants were older than 39 years old, were actively using the BHR platform within the last 6 months and consented to the study. Those eligible participants signed a consent form, approved by the University of Pennsylvania and UCSF IRB, accepting sharing of data between BHR and Monell. Once consented, the BHR group gave participants a participation code and Monell’s contact email to inquire about the study.

Interested participants provided a mailing address and received a letter that contained the 9 odor cards packed together in a small plastic bag along with instructions. The provided instructions included: the link to the website that would record their responses, their study code, and instructions on how to sample the odors and make their online response.

**Fig 1:**
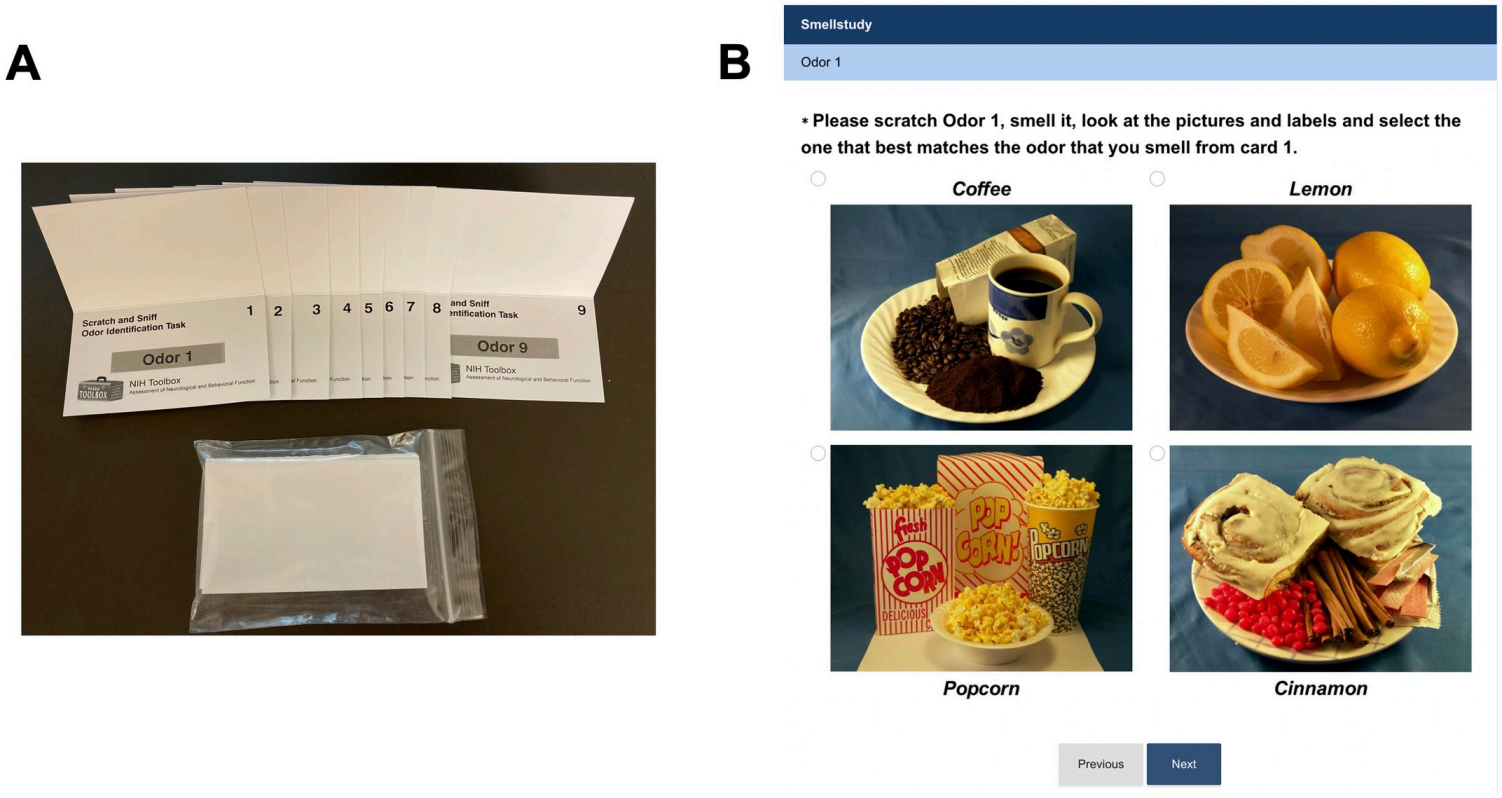
NIH Toolbox Odor ID Test. A) Modified odor identification cards, each one has a cover to prevent odor cross-contamination. All nine folded odor identification cards were mailed together inside a small plastic bag B) Screenshot of the online platform showing the four possible answers for odor 1.

**Fig 2:**
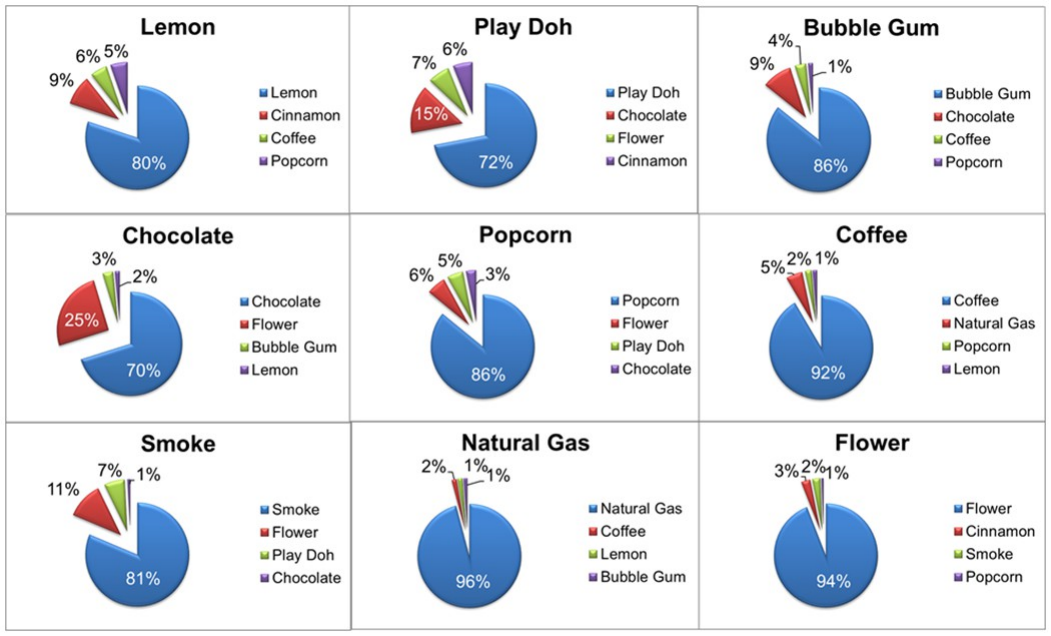
Odor identification attributed to each odor card. The correct response is at the header of each panel. Legend in each figure indicates the 4 options from which the participant had to choose, one was the correct answer and the other 3 were the distractors.

Participants self-administered the test and recorded their answers on a secure online survey platform (www.surveymonkey.com). For each odor card, the online survey presented four possible odor identification responses as pictures with labels in English; only one was the target/correct odor identifier. To self-administer the stimulus, the participant was instructed to use a paper clip to scratch the micro-encapsulated odor patch on the card and smell the released odor. Responses were made by clicking on the chosen picture that corresponded to the odor card being smelled, thus mimicking the way this test was originally performed. Feedback on the accuracy of their response was not provided. The NIH Toolbox Odor ID Test and a screenshot of the website are shown in Fig 1. The nine odors used in the NIH Toolbox Odor ID test are: Lemon, Play Doh, Bubble Gum, Chocolate, Popcorn, Coffee, Smoke, Natural Gas, and Flower. The 3 distractors used for each odor card are described as the legend of each graph in Fig 2.

### Statistical analysis

Data were analyzed used TIBCO Statistica software version 13.5. The percentage out of 9 possible accurate odor responses was the dependent variable, and grouped participants into decades. To evaluate the well-known gender disparity in odor identification, we performed an ANCOVA using gender as factor. To analyze age-associated olfactory decline we used a one-way ANOVA. Effect size for gender was calculated using Hedges’ g statistic. For comparison of individual odor cards accuracy, we used a MANOVA with posthoc analysis using Tukey unequal n. For the age group comparison we used an ANOVA. The norming NIH Toolbox dataset used for the validation of the NIH Toolbox can be retrieved online. The NIH odor ID scores can be obtained at [21] and the correct answer for each card at [22]. The data (Monell, and NIH Toolbox data) used for analysis can be found in the S1 Data.

## Results

For this study, we used the responses of the 1,163 participants who completed the test and could be identified (see participants’ demographic information in Table 1). A total of 3,236 subjects received an email from the BHR recruiting for the smell study. About 57%, 1,841 participants showed interest in participating in the smell study. Of those, 76.75% (N = 1,413) contacted Monell and provided a mailing address, and 82.73% (N = 1,169) of those who received the odor identification test signed into the online platform, gave consent, and started the odor test. Three participants used incorrect codes, making it impossible to identify them and three additional participants did not complete the test, claiming that they had problems using the website and/or had problems identifying the odors and voluntarily withdrew from the study, which brought the total from 1169 to 1163. Of those, six participants did the test twice due to problems with the odor cards or internet connection (their second response is the one chosen for analysis). Overall, our participants comprised a healthy group with only a few conditions that could be expected to result in olfactory dysfunction. Out of 1163 participants, only 46 indicated they had motor neuron disorder (e.g. Parkinson’s), 36 indicated that they had mild cognitive disorder (e.g. Alzheimer’s) and 20 indicated they had a prior traumatic brain injury.

**Table 1:** Participants’ demographic information. The population ranged from 40 to 90 years old. A total of 1,163 participants completed this study.

Gender		Race		Ethnicity		Age Distribution	
Male	287	Caucasian	1089	Not Latino	1117	40-49	38
Female	876	Asian	20	Latino	20	50-59	300
		African American	10	No answer	26	60-69	537
		Mixed	27			70-79	266
		Pacific Islander	1			80-90	22
		Native American	4				
		Other	9				
		No answer	3				

As shown in Fig 2, we found that odor integrity appeared to be maintained during the mailing process. Natural gas odor was the easiest to identify by 96% of the participants while chocolate was only correctly identified by 70% of the participants with 25% of the sample confusing it with a flower odor.

In order to compare our results with normative data collected on the NIH Toolbox as well as look at age differences, we used two different age stratifications both used in the normative data for the NIH Toolbox. First, to look at differences between age and individual cards, we used the NIH Toolbox grouping of: 1) younger than 65 years old and 2) 65 years old and older. Second, for a more high-resolution look at how overall performance differed between age groups, we used the NIH Toolbox stratification of decades starting at 40 years old (i.e., 40-49, 50-59, 60-69, 70-79) with the 80-year-old category ending at 85 years old. (For this reason 2 of our BHR participants who were older than 85 years were removed from these analysis [N = 1161]).

When looking at performance on each individual card, as broken down by age, we see few significant differences (Lemon, Play Doh, and Smoke for self-administered (F(8,9128)=25.9, p<0.001); Play Doh and Flower for the tester-administered (F(8,6888)=22.2, p<0.001). Accuracy for some odors like Natural Gas, Coffee, or Flower appeared to be better while others such as Play Doh, Chocolate and Smoke seem to be more difficult to identify (Table 2), data obtained via remote self-administration was comparable to data obtained when tester-administered.

**Table 2:** Comparison of correct responses obtained performing the test via self-administration or with a tester administering the test. A) Percentage of individuals younger than 65 that correctly identified the odorant. B) Percentage of individuals older than 65 that correctly identified the odorant. Significance determined by Tukey unequal n posthoc.

A	Self-administered	Tester-administered	p	B	Self-administered	Tester-administered	p
years (n.test)	40-64 (597)	40-64 (546)		years (n.test)	65-85 (564)	65-85 (299)	
**Lemon**	86.1	95.2	<.001	**Lemon**	76.4	87.0	ns
**Play Doh**	84.1	61.4	<.001	**Play Doh**	59.2	24.7	<.001
**Bubble Gum**	88.3	93.4	ns	**Bubble Gum**	83.0	77.9	ns
**Chocolate**	70.4	71.6	ns	**Chocolate**	70.2	64.9	ns
**Popcorn**	87.4	84.1	ns	**Popcorn**	84.2	81.9	ns
**Coffee**	94.1	94.3	ns	**Coffee**	88.7	90.0	ns
**Smoke**	84.9	74.2	<.001	**Smoke**	77.5	66.6	ns
**Natural Gas**	97.7	96.9	ns	**Natural Gas**	94.0	88.6	ns
**Flower**	96.0	90.5	ns	**Flower**	92.0	78.6	<.01

Self-administration of the test reliably detected the known gender-associated olfactory ability effect, in which females are shown to perform with higher accuracy on an odor identification test. Accuracy was higher for women compared to men after controlling for participants’ age, F(1, 1160) = 22.953, *p* <.0001. The effect size calculated as Hedge’s g, was 0.41.

**Fig 3:**
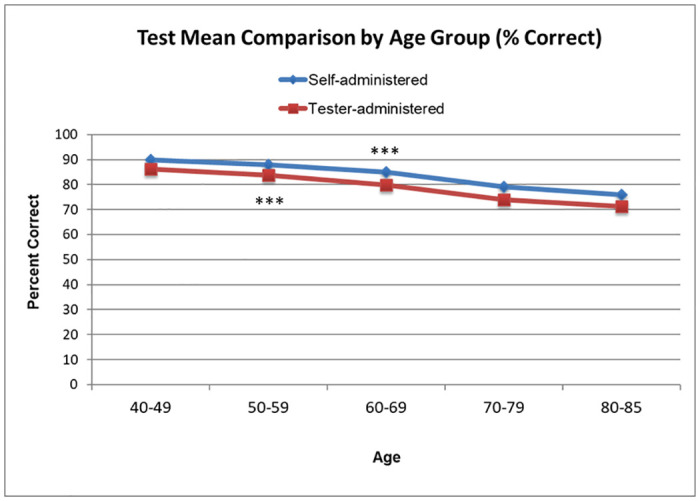
Comparison of the self-administered vs tester-administered NIH Toolbox test. Mean proportion correct score for the self-administered test (solid line) compared to the tester-administered test (dashed line). Age-dependent olfactory decline can be detected using the NIH odor identification cards. For the self-administered group 40-49 n = 38, 50-59 n = 300, 60-69 n = 537, 70-79 n = 266, 80-85 n = 20. For the tester-administered group 40-49 n = 256, 50-59 n = 192, 60-69 n = 158, 70-79 n = 143, 80-85 n = 96. ***Significance p<0.001.

Self-administration of this test also replicated the age-related loss in olfactory acuity, F(4, 1156)=14.564, p<.0001 that was found in the tester-administered test F(4, 841)=23.929, p<0.0001 an important outcome since it was possible that unsupervised participants may have elicited help from younger individuals or those with better olfactory function. We clustered participants into 5 decade-groups consistent with the age distribution performed when the test was administered in a testing facility (Fig 3). We saw a significant difference between self- and tester-administered NIH Toolbox scores, such that scores on self-administered tests were significantly higher in the “50-59” group (F(1,490)=12.0, p<0.001) as well as the “60-69” group (F(1,649)=12.7, p<0.001) (Fig 3).

## Discussion

In this study, we explored the feasibility of using the NIH Toolbox Odor ID Test as an inexpensive and convenient method to test olfactory performance when self-administered. The NIH Toolbox Odor ID test has been validated against the current industry and clinical standards (i.e., UPSIT and B-SIT) and has been shown to perform similarly across gender and age brackets [18]. From recent studies, a picture has emerged indicating that measuring olfaction has widespread utility since it seems to be a very good indicator of overall health and a potential correlate of cognitive dysfunction.

Further, and more importantly, the widespread impact of the SARS-COV-2 virus on smell loss [23] has shown the urgent need for self-administered, inexpensive smell tests. Several approaches have been used to test the sense of smell at home using common household products to test participants’ olfactory performance [24] or development of new rapid olfactory tests like the SCENTINEL that can be easily mailed [25], each with their own strengths and weakness (consistency of stimuli for household products, and number of stimuli in SCENTINEL). We feel the NIH Toolbox overcomes both of these obstacles by having a standard variety of stimuli that perform as well existing tests for a fraction of the cost.

One of our main concerns was that shipping the nine odor cards together could cause a cross-contamination problem. When the test was administered in the research setting, the scratch and sniff cards were segregated into different packets, each one containing the same odor card. To minimize the potential for contamination, we added a flap to cover each odor card. We recognized that cross-contamination is still possible when mailing the 9 odor cards together. However, based on the results obtained from the unsupervised test, which are comparable to the ones obtained during tester administration, we believe that cross-contamination did not have a significant impact on test performance.

Our results are in accordance with the existing literature as it relates to gender and age differences in olfactory ability. In accordance with a meta-analysis using data from 106 papers seems to confirm that women have a better sense of smell compared to males [26], we detected a gender-associated olfactory ability effect in the self-administered test with an effect size very similar to that reported in Sorokowski, et al (2019). Further, in literature looking at olfactory ability over age cohorts we see a consensus that there is a decline in ability over time [13, 18] a trend reflected above in the NIH Toolbox data and replicated by our self-administered test with younger participants performing better than older participants.

In sum, the importance of olfactory testing has been thrust into the spotlight because of the COVID pandemic. The existing tests are either expensive (BSIT, UPSIT), inconsistent (household odor models), or limited in stimuli (SCENTINEL). The NIH Toolbox has previously demonstrated itself as an inexpensive, consistent, and robust alternative to existing standardized test and we’ve currently demonstrated that its efficacy is maintained whether it is administered by a tester or self-administered.

Based on these results, we believe that the NIH Toolbox Odor ID is not only appropriate for assessment of olfactory function, but also an appropriated instrument to *self-assess* olfactory function.

## Limitations

We recognize that our sample was heavily skewed toward females (75%) and Caucasian participants. However, we had a sufficient spread across the age ranges to document age-associated olfactory decline and sufficient numbers of males to document the gender-associated superiority in odor identification.

Performance on the self-administered test for the 50-59 and 60-69 age groups is better than performance obtained when tester-administered. This could be accounted to the different gender distribution (in the self-administered test 75% population describe themselves as female, while in the tester-administered group 57 % population was described as female) and number of participants in each group, or that people doing the test unsupervised might have asked for help. We find reassuring that the test performed at home followed the pattern that shows a decrease in the test score as age increases.

## Conclusion

Results from this study revealed that the NIH Toolbox Odor ID Test can be self-administered with comparable results to normative data obtained when tester-administered in a research setting. Importantly, self-administration of the NIH Toolbox Odor ID Test was sufficiently sensitive to detect age-associated olfactory decline and gender-associated olfactory ability. Given the increased importance of measuring olfaction both acutely and across the lifespan, this study provides evidence that the NIH Toolbox Odor Identification test and other such assessments can be self-administered and distributed remotely, thus removing one of the barriers to a population assessment of olfactory function.

## Supporting information

S1 Data (XLSX)
